# Bioequivalence of levamlodipine besylate tablets in healthy Chinese subjects: a single-dose and two-period crossover randomized study

**DOI:** 10.1186/s40360-020-00459-6

**Published:** 2020-11-19

**Authors:** Xin Li, Chenjing Wang, Ting Li, Yanping Liu, Shuqin Liu, Ye Tao, Yaping Ma, Xiaomeng Gao, Yu Cao

**Affiliations:** Phase I Clinical Research Center, The Affiliated Hospital of Qingdao University, Qingdao, 266003 China

**Keywords:** Levamlodipine, Amlodipine, Bioequivalence, Pharmacokinetics

## Abstract

**Background:**

Levamlodipine, a calcium channel blocker, has been show act as a cardiovascular drug. To compare the pharmacokinetic parameters between levamlodipine (test formulation) at a single dose of 5 mg and amlodipine (reference formulation) at a single dose of 10 mg, the bioequivalence study was carried out.

**Methods:**

A single-dose randomized, open-label, two-period crossover study was designed in healthy Chinese subjects. 48 subjects were divided into fasted and fed groups equally. The subjects randomly received the test or reference formulations at the rate of 1:1. Following a 21-day washout period, the alternative formulations were received. The blood samples were collected at 1, 2, 3, 4, 5, 6, 7, 8, 9, 10, 11, 12, 14, 24, 36, 48, 72, 96, 120, 144, 168 h later. Liquid chromatography-tandem mass spectrometry (LC-MS/MS) was applied to determine the plasma concentrations of levamlodipine. Adverse events were recorded.

**Results:**

The 90% confidence intervals (CIs) of the ratio of geometric means (GMRs) of C_max_, AUC_0-t_, and AUC_0-∞_ under both fasted and fed conditions were within the prespecified bioequivalence limits between 80 ~ 125%. Under fasted conditions, 24 subjects were enrolled and completed the study. The mean C_max_ was (2.70 ± 0.49) ng/mL, AUC_0-t_ was (141.32 ± 36.24) ng × h/mL and AUC_0-∞_ was (157.14 ± 45.65) ng × h/mL after a single dose of 5 mg levamlodipine. The mean C_max_ was (2.83 ± 0.52) ng/mL, AUC_0-t_ was (153.62 ± 33.96) ng × h/mL and AUC_0-∞_ was (173.05 ± 41.78) ng × h/mL after a single dose of 10 mg amlodipine. Under fed conditions, 24 subjects were enrolled and completed the study. The mean C_max_ was (2.73 ± 0.55) ng/mL, AUC_0-t_ was (166.93 ± 49.96) ng × h/mL and AUC_0-∞_ was (190.99 ± 70.89) ng × h/mL after a single dose of 5 mg levamlodipine. The mean C_max_ was (2.87 ± 0.81) ng/mL AUC_0-t_ was (165.46 ± 43.58) ng × h/mL and AUC_0-∞_ was (189.51 ± 64.70) ng × h/mL after a single dose of 10 mg amlodipine. Serious adverse event was not observed.

**Conclusion:**

The trial confirmed that levamlodipine at a single dose of 5 mg and amlodipine at a single dose of 10 mg were bioequivalent under both fasted condition and fed condition.

**Trial registration:**

Cinicaltrials, NCT04411875. Registered 3 June 2020 - Retrospectively registered

**Supplementary Information:**

The online version contains supplementary material available at 10.1186/s40360-020-00459-6.

## Background

Hypertension is one of the most common risk factors of cardiovascular disease and stroke, which can lead to serious complications [1]. Amlodipine, a dihydropyridine calcium antagonist, has therapeutic effect on hypertension and angina pectoris. Amlodipine acted by inhibiting the influx of calcium through L-type calcium channels into vascular smooth muscle cells, preventing vasoconstriction while simultaneously improving blood flow. Usually, amlodipine is prescribed at a daily dose of 5 and 10 mg, may reach bioavailability of 60 ~ 90% [2]..Amlodipine is an racemic mixture, including (R)-amlodipine and (S)-amlodipine, but only the latter has therapeutic activity [3]. (S)-amlodipine, known as levamlodipine, similar to amlodipine in pharmacology, also play a role in vasodilation and decreasing blood pressure [4, 5].

Pharmacokinetics of levamlodipine besylate 2.5-mg tablet in healthy male subjects was studied previously [6]. However, pharmacokinetic studies for other doses of levamlodipine have not been fully carried out yet. Furthermore, the bioequivalence between levamlodipine and amlodipine has never been verified. The bioequivalence study was designed to compare the pharmacokinetic parameters between levamlodipine at a single dose of 5 mg and amlodipine at a single dose of 10 mg.

## Methods

### Ethics

The trial was performed abiding by the Declaration of Helsinki [7], Good clinical practice (GCP) [8] and the guidelines of China National Medical Products Administration (NMPA). Relevant documents, including protocol, informed consent and drug inspection report were all approved independently by the Medical Ethics Committee of the Affiliated Hospital of Qingdao University (No.: QYFYEC 2018–065-01). All protocol violations have been reported to the Medical Ethics Committee.

### Subjects

The inclusion criteria for the volunteers included as follows: 1) Healthy male and female aged 18 and above. 2) The body mass index is in the range of 18.6 ~ 28.5 kg/m^2^ (including the boundary value). The weight of male is not less than 50.0 kg, and that of female is not less than 45.0 kg. 3) The following inspection indexs are normal and abnormal without clinical significance. The inspection including: vital signs, physical examination, blood routine, blood biochemistry, urinalysis, serological tests for hepatitis B virus, hepatitis C virus, human immunodeficiency virus (HIV), and syphilis virus, 12-lead electrocardiogram (ECG), breath test for alcohol, drug abuse test, pregnancy test for female. 4) The subjects have no family planning within 3 months and could select contraceptive method. 5) Before the study, all subjects have been informed of the study’s purpose, protocal, benefits and risks, and signed the informed consent voluntarily.

The exclusion criteria included as follows: Being allergy to the study medications, smoking, alcohol abuse, and participation in another clinical trial within 3 months.

### Study design

The single-dose randomized, open-label, two-period crossover study was executed in the Phase I Clinical Research Center of the Affiliated Hospital of Qingdao University. According to the random table generated by SAS 9.4, the subjects were divided into four groups (Table 1). The qualified volunteers were hospitalized in the Phase I Clinical Research Center, and fasted for 10 h overnight until administration. Levamlodipine at a single dose of 5 mg or amlodipine at a single dose of 10 mg was swallowed with 240 ml water at room temperature. 4 mL blood samples were taken before administration and at 1, 2, 3, 4, 5, 6, 7, 8, 9, 10, 11, 12, 14, 24, 36, 48, 72, 96, 120, 144, 168 h after administration. The samples were centrifuged at 1800 g for 10 min at 4 °C to separate the plasma. The plasma samples were divided into two aliquots and stored at − 80 °C until bioanalysis. Since the half-life of levamlodipine is about 30 ~ 50 h, washout period, the interval between two administration, was set at 21 days. The operation of the two periods was consistent. Moreover, in the fed group, the high-fat breakfast was arranged within half an hour before taking the medicine.

**Table 1:** Study design for the bioequivalence evaluation of levamlodipine

Groups	Number of cases	The first period	The second period
Fasted group	12	T ^a^	R^b^
	12	R	T
Fed group	12	T	R
	12	R	T

^a^T: test formulation, levamlodipine besylate, 5 mg /tablet, 1 tablet;

^b^R: reference formulation, amlodipine besylate, 10 mg /tablet, 1 tablet

### Safety assessment

The safety was assessed by monitoring vital signs and laboratory tests. Vital signs, such as body temperature, blood pressure, and heart rate, were measured before administration and at 3, 8, 24, 36, 48, 72, 96, 120, 144, 168 h after administration. Before removal from this study, the subjects were evaluated with blood routine, blood biochemistry, urinalysis, pregnancy test for female, and 12-lead ECG. For the adverse events (AEs), clinical symptoms, severity, occurrence and ending time, duration, treatment measures and the correlation with the drugs were recorded. All of the AEs that occur within the 7 half lives of the drug were recorded and followed up, unless the subjects returned to normal or stable, or failed to visit.

### Bioanalysis

The analysts were blinded to the randomization. Plasma samples were determined by the liquid chromatography-tandem mass spectrometry (LC-MS/MS), which was tested by Suzhou Shenglin Pharmaceutical Technology Co., Ltd. An ACQUITY ultra-high-performance liquid chromatography unit (SHIMADZU, Nexera UHPLC LC-30A, Japan) and a mass spectrometer (Applied Biosystems, MDS Sciex, Triple Quad 6500 plus, Concord, Canada) were used in the study. Under multiple reaction monitoring, LC-MS/MS system adopts positive ionization mode. Data collection and analysis was employed with Analyst 1.6.3 software (Applied Biosystems, Foster City, CA, U.S.A.). The solid-phase extraction (SPE) experiments [9] were performed by HLB 96-well Plate (Waters Oasis, WAT058951). The plate contains a reversed phase functionalized polymeric sorbent (30 mg/well), in which particle size is 30 μm.

**Fig. 1:**
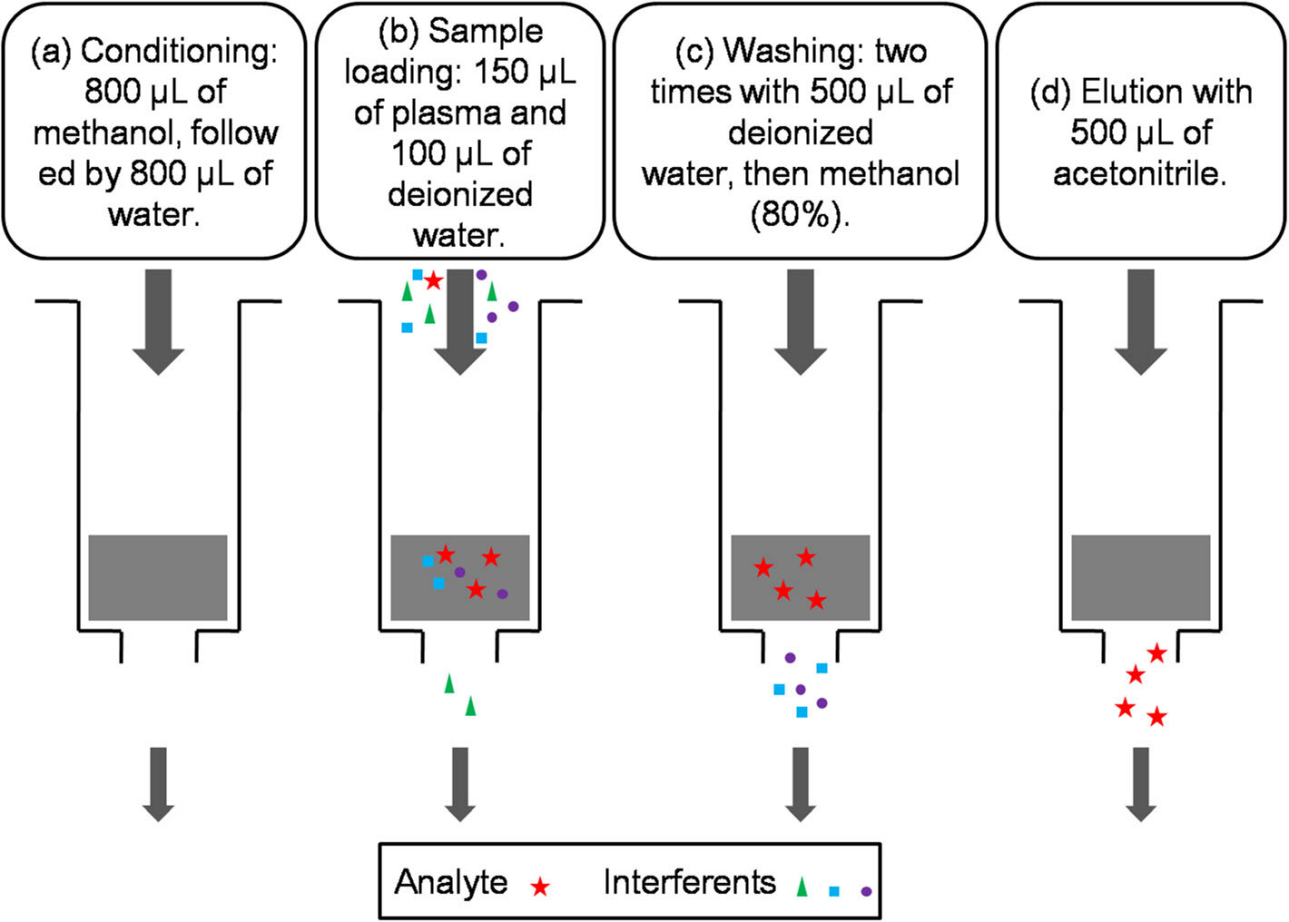
The process of the cleaning and extraction of analytes

Figure 1 summarized the process of the cleaning and extraction. The cartridges were activated by 800 μL of methanol and cleaned by 800 μL of deionized water. The column was loaded with 150 μL of plasma sample or calibrator, 50 μL of working fluid (5 ng/mL) and 100 μL of deionized water. Clean the column twice with 500 μL deionized water. The column was depolarized with 80% methanol and washed twice with 500 μL pure acetonitrile to elute levamlodipine. Dry the solution with the pure nitrogen stream. 150 μL of pure acetonitrile was add into each sample, and mixed at room temperature for 10 min. Finally, 20 μL of the sample was injected into the LC-MS / MS system.

The method is verified fully by selectivity, accuracy, precision, calibration curve and stability. The drug concentration was linear within the range of 0.05 ~ 10.0 μg × L^− 1^. The lower limit of quantification was 0.05 μg × L^− 1^, and the equation was Y = 0.55667X - 0.0030182 (r^2^ = 0.9949). The intra- and interday maximum precision was 5.4 and 4.8%, respectively. The intra- and interday accuracy was − 6.7 ~ 3.9% and − 3.3 ~ 3.3%, respectively. The extraction recovery of levamlodipine was 94.7 ± 3.9%. There was no significant interference in selectivity and stability.

### Pharmacokinetic analysis

All subjects completed the study and the data were included in the pharmacokinetic analysis. The pharmacokinetic parameters were calculated according to non-compartment model with Phoenix™ WinNonlin® 8.0 software (Pharsight, St. Louis, MO, USA). The value below the lower limit of quantification that occur before the first measurable concentration were set as zero. Subsequent values below the lower limit of quantification were excluded. The primary PK parameters were the maximum plasma concentration (C_max_), the area under the plasma concentration-time curve from 0 to the last measured time point (AUC_0-t_), and the area under the plasma concentration-time curve from 0 to infinity (AUC_0-∞_). The secondary PK parameters were the observed time to C_max_ (T_max_) and the apparent terminal half-life (T_1/2_).

### Statistical analysis

Analysis of variance (ANOVA) was performed on the logarithmically transformed C_max_, AUC_0-t_, and AUC_0-∞_ to assess the effects from subject, treatment, period, and preparation. Statistical data were presented as mean ± standard deviation (SD). The probability value less than 0.05 is considered statistically significant. The GMRs of the primary PK parameters and their 90% confidence intervals (CIs) were calculated. If it is within the equivalent range (80 ~ 125%), it is judged as bioequivalence, and the results of double unilateral t-test are listed. T_max_ was analyzed by non-parametric statistical test. Statistical analyses were performed by SAS 9.4 (SAS Institute Inc. Cary, NC, USA).

## Results

### Characteristics of the subjects

All subjects completed the study. A total of 24 subjects including 6 women and 18 men enrolled in the fasted group. Among them, 23 were Han and 1 was Manchu nationality. Parameter, mean ± SD (range): age, 31.04 ± 8.04 years (19.00 ~ 48.00 years); weight, 62.06 ± 8.59 kg (48.00 ~ 83.00 kg); height, 168.27 ± 7.97 cm (152.00 ~ 185.00 cm); body mass index (BMI), 21.90 ± 2.57 kg × m^− 2^ (19.00 ~ 27.90 kg × m^− 2^). A total of 24 subjects including 6 women and 18 men enrolled in the fed group. All of them were Han nationality. Parameter, mean ± SD (range): age, 32.46 ± 10.45 years (18.00 ~ 51.00 years); weight, 66.88 ± 8.8 kg (50.00 ~ 81.00 kg); height, 167.77 ± 6.63 cm (154.00 ~ 178.00 cm); BMI, 23.76 ± 2.86 kg × m^− 2^ (18.90 ~ 28.30 kg × m^− 2^).

### Pharmacokinetics

**Fig. 2:**
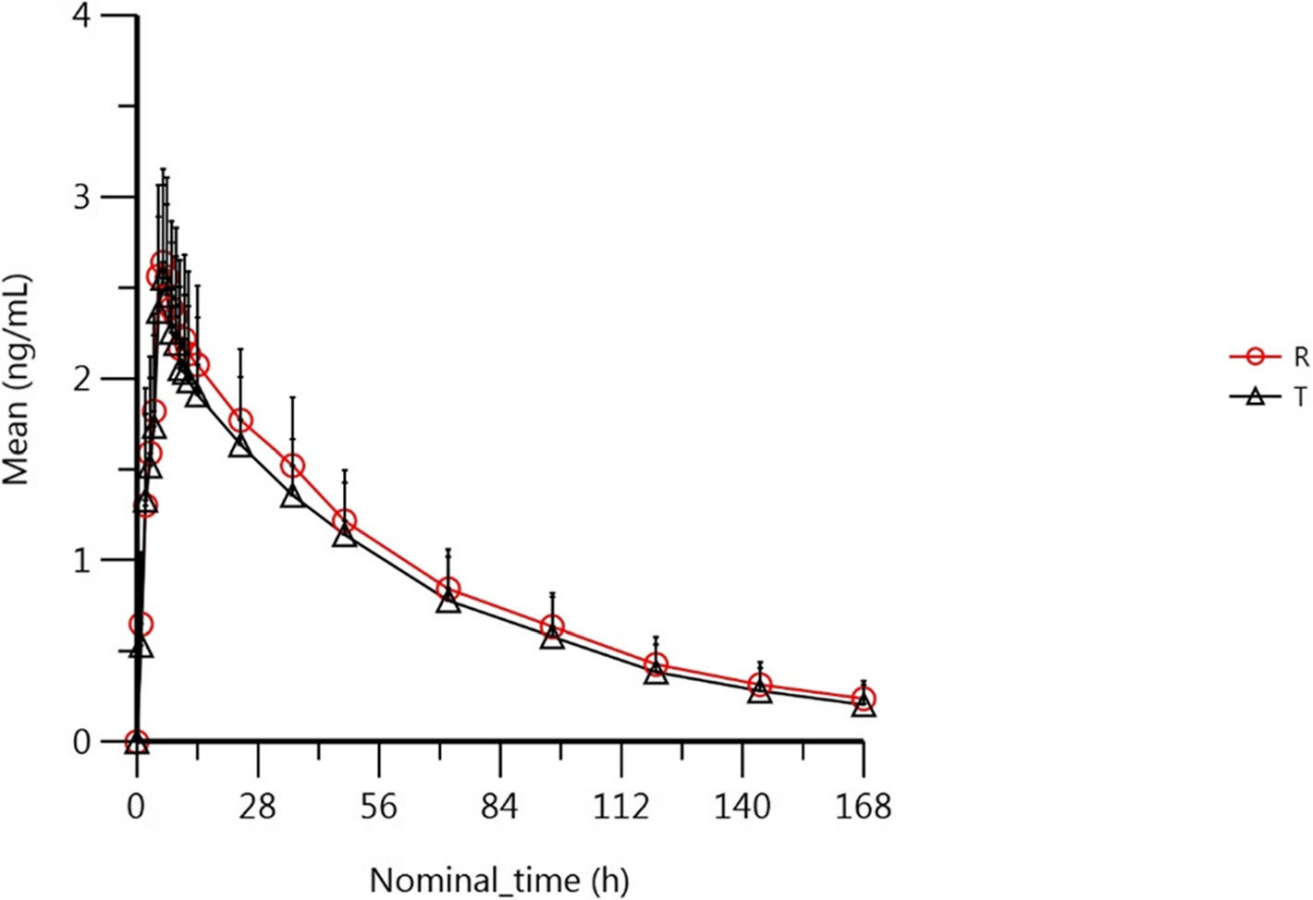
The mean plasma concentration-time curves following a single dose of test formulations or reference formulations in the fasted group

**Fig. 3:**
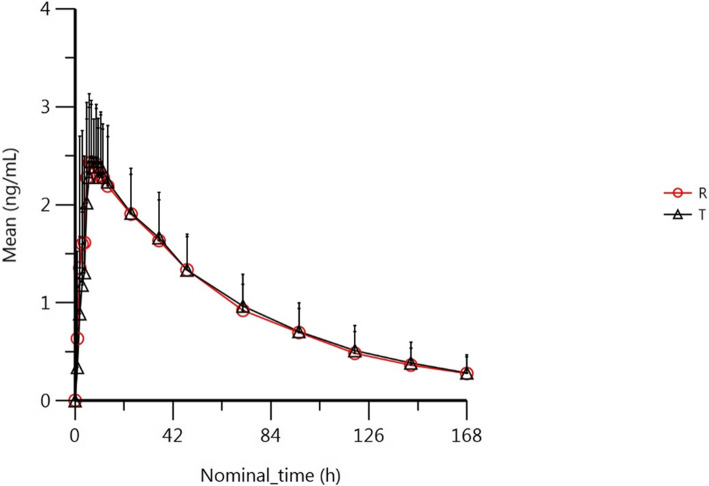
The mean plasma concentration-time curves following a single dose of test formulations or reference formulations in the fed group

Following a single dose of test formulations or reference formulations, the mean plasma concentration-time curves were shown in Fig. 2 (the fasted group) and Fig. 3 (the fed group). Individual plasma concentration-time data of levamlodipine was show supplementary file.

Mean pharmacokinetic parameters from the fasted group (Table 2) and the fed group (Table 3) are summarized. In the fasted group (Table 4), ANOVA for C_max_, AUC_0-t_, and AUC_0-∞_ indicated a lack of effects on treatment. ANOVA for C_max_ indicated a significant difference in subjects (*p* ≤ 0.05). ANOVA for AUC_0-t_ (*p* ≤ 0.05) and AUC_0-∞_ (*p* ≤ 0.05) indicated a significant difference in formulations and preparations. In the fed group (Table 5), ANOVA for C_max_, AUC_0-t_, and AUC_0-∞_ indicated a lack of effects in treatments and preparations. ANOVA for C_max_ (*p* ≤ 0.05), AUC_0-t_ (*p* ≤ 0.05), and AUC_0-∞_ (*p* ≤ 0.05) indicated a significant difference in subjects. ANOVA for AUC_0-t_ indicated a significant difference in periods (*p* ≤ 0.05).

**Table 2:** Pharmacokinetic parameters of levamlodipine test and reference formulations in the fasted group (*n* = 24)

Parameter	Arithmetic mean ± SD (%CV)^a^	
	T	R
C_max_/μg × L^−1^	2.70 ± 0.490 (18.2%)	2.83 ± 0.516 (18.2%)
AUC_0–t_/μg × h × L^− 1^	141.32 ± 36.24 (25.65%)	153.62 ± 33.96 (22.10%)
AUC_0–∞_/μg × h × L^− 1^	157.14 ± 45.65 (29.05%)	173.05 ± 41.78 (24.15%)
T_1/2_/h	49.46 ± 11.35 (22.95%)	52.92 ± 14.51 (27.42%)
t_max_*/h	6.00 (2.00, 8.00)	6.00 (3.00, 12.00)

^a^All values are represented as arithmetic mean ± standard deviation (CV, %) with [geometric mean] unless otherwise specified. *tmax is shown as median (minimum-maximum)

**Table 3:** Pharmacokinetic parameters of levamlodipine test and reference formulations in the fed group (*n* = 24)

Parameter	Arithmetic mean ± SD (%CV)^a^	
	T	R
C_max_/μg × L^−1^	2.73 ± 0.547 (20.1%)	2.87 ± 0.812 (28.3%)
AUC_0–t_/μg × h × L^− 1^	166.93 ± 49.96 (29.93%)	165.46 ± 43.58 (26.34%)
AUC_0–∞_/μg × h × L^− 1^	190.99 ± 70.89 (37.12%)	189.51 ± 64.70 (34.14%)
T_1/2_/h	53.38 ± 10.99 (20.58%)	53.56 ± 13.41 (25.05%)
t_max_*/h	7.00 (5.00, 14.00)	7.00 (2.00, 24.00)

^a^All values are represented as arithmetic mean ± standard deviation (CV, %) with [geometric mean] unless otherwise specified. *tmax is shown as median (minimum-maximum)

**Table 4:** ANOVA results of main pharmacokinetic parameters in the fasted group

Dependent variable	Subject		Treatment		Period		Preparation	
	F ^a^	P ^b^	F	P	F	P	F	P
LnC_max_	2.91	***0.0077***	3.90	0.0610	0.84	0.3686	1.64	0.2132
LnAUC_0–t_	18.55	***0.0000***	1.35	0.2578	11.21	***0.0029***	16.52	***0.0005***
LnAUC_0–∞_	22.19	***0.0000***	0.58	0.4540	9.95	***0.0046***	21.16	***0.0001***

^a^F = Fisher value

^b^P = probability value

**Table 5:** ANOVA results of main pharmacokinetic parameters in the fed group

Dependent variable	Subject		Treatment		Period		Preparation	
	F ^a^	P ^b^	F	P	F	P	F	P
LnC_max_	6.49	***0.0000***	0.01	0.9110	1.22	0.2819	1.08	0.3101
LnAUC_0–t_	13.50	***0.0000***	0.11	0.7425	4.91	***0.0373***	0.01	0.9302
LnAUC_0–∞_	15.68	***0.0000***	0.04	0.8485	3.51	0.0745	0.01	0.9404

^a^F = Fisher value

^b^P = probability value

The 90% CIs for the GMRs of C_max_, AUC_0-t_, AUC_0-∞_ and the power were presented in Table 6 (the fasted group) and Table 7 (the fed group). These ratios were within the predefined equivalence limit of 80 ~ 125%.

**Table 6:** 90% CIs for the geometric mean ratios of C_max_, AUC_0–t_, and AUC_0–∞_ in the fasted group (*n =* 24)

Parameter	T/R (%)	90% CIs	Power (%)
C_max_	95.41	89.59–101.61%	99.87
AUC_0–t_	91.28	87.83–94.87%	100.00
AUC_0–∞_	89.81	86.28–93.49%	99.92

**Table 7:** 90% CIs for the geometric mean ratios of C_max_, AUC_0–t_, and AUC_0–∞_ in the fed group (*n* = 24)

Parameter	T/R (%)	90% CIs	Power (%)
C_max_	96.48	90.93–102.37%	99.98
AUC_0–t_	100.24	95.75–104.93%	100.00
AUC_0–∞_	100.22	95.36–105.33%	100.00

### Safety

During the whole study period, both test preparation and reference preparation showed good tolerance. The AEs found in physical examination, 12-lead ECG and laboratory examination were listed in Table 8 (the fasted group) and Table 9 (the fed group). None of them were judged as serious adverse events (SAEs).

**Table 8:** Adverse events in the fasted group

Random number	AEs	Treatment	Relationship with the formulations
K002	Cholesterol rise	R	Probably related
K003	Increased RBC count in urine	T	Probably related
K004	Urinary leukocyte positive	R	Probably related
K006	Toothache	R	Probably related
K011	Anemia	R	Probably related
K012	Leukocyte count increased	T	Probably related
	Toothache	T	Probably related
	Neutrophil count increased	T	Probably related
K017	Anemia	R	Probably related
K021	Triglyceride rise	R	Probably related
K022	Rhinorrhea	R	Probably related
K023	Trauma of right foot	R	Definitely not

**Table 9:** Adverse events in the fed group

Random number	AEs	Treatment	Relationship with the formulations
F003	Vasovagal response	T	May be irrelevant
	Toothache	R	Probably related
F010	New degree I atrioventricular block	T	Probably related
F014	Vasovagal response	T	May be irrelevant
	Triglyceride rise	R	Probably related
F015	Triglyceride rise	T	Probably related
F017	Triglyceride rise	R	Probably related
F019	Prolonged APTT ^a^	T	Probably related

^a^*APTT* activated partial thromboplastin time

## Discussion

On March 5, 2016, the general office of the State Council of the people’s Republic of China issued opinions on the quality and efficacy consistency evaluation of generic drugs. Accordingly, the present study was performed to compare the pharmacokinetics of a newly-developed levamlodipine besylate tablet at a single dose of 5 mg (test formulation, anglikang Pharmaceutical Company Co., Ltd., Zhejiang, China) with that of a marketed amlodipine besylate tablet at a single dose of 10 mg (reference formulation, Pfizer Pharmaceuticals Limited, USA) for assessment of bioequivalence in healthy Chinese volunteers under fasted and fed conditions. The two medicinal products are bioequivalent when their 90% CI of the AUC_0-t_, AUC_0-∞_ and C_max_ of the reference preparation over the test preparation fall between the pre-determined limits of 80 ~ 125%. The two medicinal formulations were well tolerated. No subject withdrew from the study due to any AEs, and no SAEs occurred.

Amlodipine besylate tablets were developed by Pfizer Pharmaceutical Co., Ltd., in which the ratio of (R)-amlodipine to (S)-amlodipine is 1:1. The active component of amlodipine is (S)-amlodipine, also known as levamlodipine. In vivo, there is no mutual transformation between (R)-amlodipine and (S)-amlodipine. Before this study (November 13, 2019), there is no approved levamlodipine besylate tablet in the European Union, the United States, Japan, and China. Moreover, the pharmaceutical research of the the new developed test formulation is carried out according to the amlodipine besylate tablets developed by Pfizer. Combined with Chinese drug consistency evaluation catalogue, amlodipine besylate tablets (Norvasc®) produced by Pfizer was selected as the reference formulation in this study. On December 19, 2019, levamlodipine maleate tablets (Conjupri®) produced by CSPC Ouyi pharmaceutical Co., Ltd. was approved by FDA. However, the acid radical of this product is different from the test formulation in this study. Therefore, NMPA approved that amlodipine besylate tablets developed by Pfizer was selected as the reference preparation in this study.

In this study, the parameters such as recoveries, matrix effects, linear range, lower limit of quantification, stability by specificity, precision, and accuracy specifications, were investigated to confirm the method of LC-MS/MS. Concentration of levamlodipine in human plasma showed good linear relationship within 0.05 ~ 10 μg × L^− 1^. The relative standard deviation (RSD) values of intra- and interday precision were both less than 10%. The matrix effect induced by endogenous interfering substances in biological samples did not affect the ionization efficiency and signal intensity of levamlodipine [9]. According to FDA bioequivalence guidance, the final elimination half-life of amlodipine is about 30 ~ 50 h. Thus, washout period is set to 21 days, which is a length of time greater than seven half lives. And the blood samples taken for 168 h was enough.

In view of the inhibitory effect of amlodipine on CYP3A4 [10], subjects with behaviors of drinking, smoking and drug intake through CYP3A4 metabolism were excluded. During the trial, the administration of drugs metabolized by CYP3A4 did not occur. As an open-label study, AEs assessment may not be objective enough. When the test formulation passes the evaluation and enters the market, the efficacy and side effects need to be further explored.

## Conclusions

The trial confirmed that levamlodipine at a single dose of 5 mg and amlodipine at a single dose of 10 mg were bioequivalent under both fasted condition and fed condition. If the test formulation levamlodipine can be approved by NMPA, it can be used in the treatment of hypertension in clinic.

## Supplementary Information

**Additional file 1.**

## Data Availability

We have shared the raw data by providing it in a supplementary file.

## Abbreviations

LC-MS/MS: Liquid chromatography-tandem mass spectrometry
CIs: Confidence intervals
GMRs: Ratio of geometric means
GCP: Good clinical practice
NMPA: China National Medical Products Administration
HIV: Human immunodeficiency virus
AEs: Adverse events
SPE: Solid-phase extraction
AUC: Area under curve
C_max_: Maximum plasma concentration
T_max_: Time to C_max_
T_1/2_: Elimination half-life
SD: Standard deviation
BMI: Body mass index
ECG: Electrocardiogram
SAEs: Serious adverse events
RSD: Relative standard deviation
