# Supplementary material for: Bioequivalence of levamlodipine besylate tablets in healthy Chinese subjects: a single-dose and two-period crossover randomized study
Source: BMC Pharmacol Toxicol. 2020 Nov 19;21:80. doi: 10.1186/s40360-020-00459-6 (PMC7678077; doi:10.1186/s40360-020-00459-6)
Supplement: Supplementary file 1 — Additional file 1. [file 40360_2020_459_MOESM1_ESM.docx]

**The Fasting Group**

**Table 1.** Individual plasma concentration-time data of levamlodipine test formulationin the fasting group (ng/ml). (Supplementary file for Fig. 2)

| SUB | SEQ | PER | GRP | TRT | C1 | C2 | C3 | C4 | C5 | C6 | C7 | C8 | C9 | C10 | C11 | C12 | C13 | C14 | C15 | C16 | C17 | C18 | C19 | C20 | C21 | C22 |
| --- | --- | --- | --- | --- | --- | --- | --- | --- | --- | --- | --- | --- | --- | --- | --- | --- | --- | --- | --- | --- | --- | --- | --- | --- | --- | --- |
| K001 | RT | 2 | RT | T | 0.00 | 1.24 | 1.78 | 1.24 | 1.55 | 2.33 | 2.00 | 2.07 | 2.07 | 1.98 | 1.74 | 1.71 | 1.63 | 1.61 | 1.15 | 0.958 | 0.723 | 0.507 | 0.383 | 0.232 | 0.156 | 0.116 |
| K002 | TR | 1 | TR | T | 0.00 | 0.524 | 1.82 | 1.52 | 1.53 | 2.95 | 3.10 | 2.98 | 2.87 | 2.74 | 2.63 | 2.53 | 2.49 | 2.45 | 2.23 | 1.84 | 1.72 | 1.08 | 0.715 | 0.476 | 0.372 | 0.247 |
| K003 | RT | 2 | RT | T | 0.00 | 0.379 | 0.952 | 1.49 | 1.74 | 2.52 | 2.68 | 2.81 | 2.30 | 2.79 | 1.98 | 2.12 | 2.24 | 2.11 | 1.83 | 1.55 | 1.46 | 1.21 | 1.02 | 0.635 | 0.512 | 0.418 |
| K004 | TR | 1 | TR | T | 0.00 | 1.24 | 1.44 | 2.16 | 1.65 | 2.00 | 2.66 | 2.19 | 1.98 | 1.86 | 1.83 | 1.74 | 1.86 | 1.92 | 1.44 | 1.17 | 0.973 | 0.595 | 0.348 | 0.218 | 0.134 | 0.0857 |
| K005 | RT | 2 | RT | T | 0.00 | 0.227 | 0.637 | 0.721 | 1.09 | 1.28 | 1.76 | 1.58 | 1.68 | 1.66 | 1.60 | 1.56 | 1.56 | 1.50 | 1.33 | 1.13 | 0.861 | 0.633 | 0.471 | 0.284 | 0.218 | 0.144 |
| K006 | TR | 1 | TR | T | 0.00 | 0.271 | 0.730 | 1.05 | 1.04 | 1.70 | 2.00 | 1.72 | 1.44 | 1.52 | 1.48 | 1.39 | 1.36 | 1.28 | 1.14 | 0.956 | 0.819 | 0.568 | 0.421 | 0.312 | 0.207 | 0.145 |
| K007 | RT | 2 | RT | T | 0.00 | 0.509 | 1.28 | 1.63 | 1.80 | 2.42 | 2.45 | 2.32 | 1.99 | 1.87 | 1.87 | 1.86 | 1.69 | 1.73 | 1.52 | 1.21 | 1.06 | 0.707 | 0.540 | 0.349 | 0.229 | 0.191 |
| K008 | TR | 1 | TR | T | 0.00 | 0.683 | 1.11 | 2.03 | 1.60 | 2.30 | 2.05 | 1.79 | 1.34 | 1.58 | 1.43 | 1.50 | 1.39 | 1.22 | 1.09 | 0.835 | 0.711 | 0.351 | 0.263 | 0.137 | 0.105 | 0.0789 |
| K009 | RT | 2 | RT | T | 0.00 | 0.619 | 1.09 | 1.48 | 1.66 | 2.63 | 3.03 | 3.37 | 3.07 | 3.07 | 2.72 | 2.64 | 2.71 | 2.54 | 2.24 | 1.86 | 1.48 | 1.09 | 0.976 | 0.622 | 0.513 | 0.448 |
| K010 | TR | 1 | TR | T | 0.00 | 0.563 | 1.63 | 1.79 | 2.25 | 2.25 | 2.98 | 2.92 | 2.77 | 2.53 | 2.20 | 2.38 | 2.47 | 2.32 | 1.99 | 1.79 | 1.40 | 0.952 | 0.665 | 0.470 | 0.343 | 0.224 |
| K011 | TR | 1 | TR | T | 0.00 | 0.853 | 1.76 | 2.00 | 2.20 | 2.83 | 2.82 | 2.72 | 2.72 | 2.36 | 2.29 | 2.31 | 2.19 | 2.13 | 2.14 | 1.49 | 1.29 | 0.847 | 0.651 | 0.410 | 0.285 | 0.191 |
| K012 | RT | 2 | RT | T | 0.00 | 0.316 | 0.396 | 1.08 | 1.48 | 2.20 | 2.28 | 2.24 | 2.11 | 2.04 | 1.65 | 1.94 | 1.92 | 1.87 | 1.73 | 1.38 | 1.26 | 0.873 | 0.746 | 0.508 | 0.408 | 0.326 |
| K013 | TR | 1 | TR | T | 0.00 | 0.335 | 1.34 | 1.41 | 1.83 | 1.91 | 2.61 | 2.05 | 2.06 | 1.98 | 2.02 | 1.97 | 2.08 | 2.10 | 1.70 | 1.53 | 1.22 | 0.829 | 0.601 | 0.402 | 0.307 | 0.213 |
| K014 | RT | 2 | RT | T | 0.00 | 0.403 | 1.43 | 1.87 | 2.28 | 3.10 | 2.92 | 2.74 | 2.61 | 2.40 | 2.02 | 1.89 | 1.78 | 1.79 | 1.34 | 1.14 | 0.838 | 0.545 | 0.398 | 0.240 | 0.169 | 0.103 |
| K015 | TR | 1 | TR | T | 0.00 | 0.581 | 1.42 | 2.10 | 2.42 | 2.96 | 2.90 | 3.10 | 2.83 | 2.73 | 2.80 | 2.64 | 2.64 | 2.93 | 2.17 | 1.87 | 1.66 | 1.25 | 1.00 | 0.691 | 0.497 | 0.371 |
| K016 | RT | 2 | RT | T | 0.00 | 0.446 | 0.604 | 1.00 | 1.39 | 2.06 | 1.83 | 1.75 | 1.63 | 1.41 | 1.30 | 1.20 | 1.22 | 1.23 | 1.08 | 0.878 | 0.710 | 0.455 | 0.365 | 0.217 | 0.151 | 0.0843 |
| K017 | TR | 1 | TR | T | 0.00 | 0.646 | 2.02 | 2.57 | 3.16 | 2.33 | 2.71 | 2.97 | 2.53 | 2.62 | 2.67 | 2.61 | 2.42 | 2.30 | 1.63 | 1.39 | 1.06 | 0.585 | 0.322 | 0.162 | 0.107 | 0.0549 |
| K018 | RT | 2 | RT | T | 0.00 | 0.439 | 1.51 | 1.56 | 1.87 | 2.14 | 2.34 | 2.55 | 2.64 | 2.40 | 2.43 | 2.24 | 2.01 | 1.81 | 1.73 | 1.46 | 1.29 | 0.962 | 0.735 | 0.503 | 0.332 | 0.236 |
| K019 | TR | 1 | TR | T | 0.00 | 0.150 | 0.653 | 1.37 | 1.33 | 2.63 | 2.68 | 2.37 | 2.14 | 1.95 | 1.82 | 1.88 | 1.82 | 1.61 | 1.34 | 1.33 | 1.03 | 0.635 | 0.433 | 0.304 | 0.238 | 0.166 |
| K020 | RT | 2 | RT | T | 0.00 | 0.528 | 2.67 | 1.33 | 1.99 | 1.92 | 2.20 | 2.43 | 2.35 | 2.12 | 2.21 | 2.05 | 1.89 | 1.84 | 1.77 | 1.39 | 1.21 | 0.795 | 0.520 | 0.393 | 0.297 | 0.219 |
| K021 | TR | 1 | TR | T | 0.00 | 0.295 | 1.07 | 1.73 | 1.46 | 2.51 | 3.25 | 2.43 | 2.21 | 2.12 | 2.13 | 2.11 | 2.07 | 1.74 | 1.46 | 1.31 | 1.16 | 0.821 | 0.513 | 0.370 | 0.294 | 0.198 |
| K022 | TR | 1 | TR | T | 0.00 | 0.615 | 1.89 | 1.11 | 1.63 | 2.43 | 2.99 | 2.79 | 2.22 | 2.40 | 2.25 | 2.05 | 2.19 | 2.14 | 1.84 | 1.41 | 1.26 | 0.906 | 0.755 | 0.528 | 0.384 | 0.286 |
| K023 | RT | 2 | RT | T | 0.00 | 0.152 | 0.277 | 0.461 | 0.729 | 1.74 | 1.52 | 1.99 | 1.59 | 1.58 | 1.48 | 1.65 | 1.65 | 1.57 | 1.33 | 1.07 | 0.901 | 0.551 | 0.404 | 0.278 | 0.141 | 0.0971 |
| K024 | RT | 2 | RT | T | 0.00 | 0.762 | 2.43 | 1.75 | 1.97 | 3.73 | 3.49 | 3.09 | 2.95 | 2.91 | 2.72 | 2.82 | 2.39 | 2.09 | 2.02 | 1.65 | 1.29 | 0.904 | 0.674 | 0.501 | 0.358 | 0.274 |
| Number of valid data | | | | | 24 | 24 | 24 | 24 | 24 | 24 | 24 | 24 | 24 | 24 | 24 | 24 | 24 | 24 | 24 | 24 | 24 | 24 | 24 | 24 | 24 | 24 |
| Mean concentration | | | | | / | 0.532 | 1.33 | 1.52 | 1.74 | 2.37 | 2.55 | 2.46 | 2.25 | 2.19 | 2.05 | 2.03 | 1.99 | 1.91 | 1.64 | 1.36 | 1.14 | 0.777 | 0.580 | 0.385 | 0.282 | 0.205 |
| SD | | | | | / | 0.285 | 0.615 | 0.485 | 0.506 | 0.521 | 0.511 | 0.499 | 0.494 | 0.475 | 0.451 | 0.429 | 0.412 | 0.425 | 0.372 | 0.308 | 0.289 | 0.241 | 0.217 | 0.153 | 0.126 | 0.108 |
| CV% | | | | | / | 53.4 | 46.2 | 31.9 | 29.1 | 22.0 | 20.0 | 20.3 | 21.9 | 21.6 | 22.0 | 21.1 | 20.7 | 22.2 | 22.8 | 22.7 | 25.4 | 31.1 | 37.5 | 39.7 | 44.7 | 52.7 |
| Maximum | | | | | / | 1.24 | 2.67 | 2.57 | 3.16 | 3.73 | 3.49 | 3.37 | 3.07 | 3.07 | 2.80 | 2.82 | 2.71 | 2.93 | 2.24 | 1.87 | 1.72 | 1.25 | 1.02 | 0.691 | 0.513 | 0.448 |
| Minimum | | | | | / | 0.150 | 0.277 | 0.461 | 0.729 | 1.28 | 1.52 | 1.58 | 1.34 | 1.41 | 1.30 | 1.20 | 1.22 | 1.22 | 1.08 | 0.835 | 0.710 | 0.351 | 0.263 | 0.137 | 0.105 | 0.0549 |

**Table 2.** Individual plasma concentration-time data of levamlodipine reference formulationin the fasting group (ng/ml). (Supplementary file for Fig. 2)

| SUB | SEQ | PER | GRP | TRT | | C1 | C2 | C3 | C4 | C5 | C6 | C7 | C8 | C9 | C10 | C11 | C12 | C13 | C14 | C15 | C16 | C17 | C18 | C19 | C20 | C21 | C22 |
| --- | --- | --- | --- | --- | --- | --- | --- | --- | --- | --- | --- | --- | --- | --- | --- | --- | --- | --- | --- | --- | --- | --- | --- | --- | --- | --- | --- |
| K001 | RT | 1 | RT | R | | 0.00 | 0.794 | 1.50 | 1.51 | 1.29 | 2.28 | 2.27 | 2.04 | 1.81 | 1.71 | 1.55 | 1.65 | 1.47 | 1.56 | 1.16 | 0.924 | 0.794 | 0.511 | 0.331 | 0.177 | 0.154 | 0.0937 |
| K002 | TR | 2 | TR | R | | 0.00 | 0.503 | 0.943 | 1.51 | 2.04 | 2.69 | 3.24 | 3.29 | 3.14 | 2.95 | 2.85 | 2.75 | 2.68 | 2.23 | 2.36 | 1.91 | 1.58 | 1.07 | 0.776 | 0.508 | 0.385 | 0.295 |
| K003 | RT | 1 | RT | R | | 0.00 | 0.833 | 2.16 | 1.82 | 2.00 | 3.02 | 2.54 | 2.57 | 2.42 | 2.43 | 2.31 | 2.25 | 2.05 | 2.14 | 1.77 | 1.46 | 1.40 | 1.05 | 0.954 | 0.754 | 0.594 | 0.454 |
| K004 | TR | 2 | TR | R | | 0.00 | 0.744 | 2.24 | 2.08 | 2.26 | 2.79 | 2.82 | 2.85 | 2.80 | 3.00 | 2.25 | 2.27 | 2.28 | 2.49 | 1.99 | 1.73 | 1.30 | 0.845 | 0.585 | 0.343 | 0.247 | 0.176 |
| K005 | RT | 1 | RT | R | | 0.00 | 0.311 | 0.554 | 0.850 | 1.11 | 1.92 | 1.78 | 1.90 | 1.78 | 1.84 | 1.66 | 1.86 | 1.74 | 1.70 | 1.39 | 1.21 | 0.980 | 0.688 | 0.539 | 0.318 | 0.239 | 0.175 |
| K006 | TR | 2 | TR | R | | 0.00 | 0.428 | 0.881 | 1.22 | 1.62 | 2.91 | 2.79 | 2.69 | 2.24 | 2.07 | 1.84 | 1.95 | 1.82 | 1.84 | 1.53 | 1.35 | 1.06 | 0.724 | 0.732 | 0.430 | 0.263 | 0.226 |
| K007 | RT | 1 | RT | R | | 0.00 | 0.462 | 0.907 | 1.34 | 1.35 | 2.59 | 2.40 | 2.44 | 2.21 | 2.44 | 1.95 | 2.02 | 1.77 | 1.95 | 1.65 | 1.50 | 1.21 | 0.892 | 0.640 | 0.409 | 0.356 | 0.253 |
| K008 | TR | 2 | TR | R | | 0.00 | 0.578 | 1.75 | 1.01 | 2.14 | 1.90 | 2.18 | 2.05 | 1.94 | 1.77 | 1.62 | 1.74 | 1.71 | 1.62 | 1.32 | 1.08 | 0.788 | 0.469 | 0.330 | 0.232 | 0.177 | 0.0984 |
| K009 | RT | 1 | RT | R | | 0.00 | 0.826 | 1.48 | 2.01 | 1.67 | 2.23 | 3.02 | 2.49 | 2.46 | 2.38 | 2.47 | 2.16 | 2.19 | 2.11 | 1.77 | 1.61 | 1.44 | 0.960 | 0.658 | 0.517 | 0.426 | 0.347 |
| K010 | TR | 2 | TR | R | | 0.00 | 0.712 | 1.51 | 2.19 | 2.72 | 3.09 | 3.10 | 3.48 | 3.21 | 3.25 | 3.06 | 2.99 | 2.75 | 2.81 | 2.30 | 1.88 | 1.40 | 0.905 | 0.628 | 0.400 | 0.264 | 0.202 |
| K011 | TR | 2 | TR | R | | 0.00 | 1.88 | 2.08 | 2.28 | 2.37 | 3.32 | 3.73 | 3.44 | 3.20 | 2.58 | 2.60 | 2.84 | 2.47 | 2.49 | 2.18 | 1.96 | 1.46 | 1.08 | 0.866 | 0.531 | 0.353 | 0.334 |
| K012 | RT | 1 | RT | R | | 0.00 | 0.330 | 1.08 | 0.979 | 1.18 | 3.04 | 2.62 | 2.42 | 2.33 | 2.14 | 1.93 | 1.99 | 1.79 | 1.96 | 1.64 | 1.25 | 1.13 | 0.760 | 0.588 | 0.410 | 0.338 | 0.292 |
| K013 | TR | 2 | TR | R | | 0.00 | 0.264 | 0.769 | 1.05 | 1.29 | 1.70 | 2.11 | 2.20 | 2.05 | 2.16 | 2.04 | 2.03 | 2.22 | 1.92 | 2.03 | 1.64 | 1.42 | 1.03 | 0.764 | 0.603 | 0.366 | 0.254 |
| K014 | RT | 1 | RT | R | | 0.00 | 1.25 | 1.69 | 2.74 | 1.75 | 2.16 | 2.37 | 2.14 | 2.04 | 1.86 | 1.70 | 1.80 | 1.61 | 1.59 | 1.24 | 1.11 | 0.857 | 0.534 | 0.388 | 0.288 | 0.186 | 0.124 |
| K015 | TR | 2 | TR | R | | 0.00 | 1.38 | 1.58 | 1.94 | 3.22 | 2.84 | 3.31 | 3.50 | 2.89 | 3.06 | 2.90 | 2.73 | 2.99 | 3.20 | 2.52 | 2.48 | 1.86 | 1.40 | 0.983 | 0.711 | 0.592 | 0.410 |
| K016 | RT | 1 | RT | R | | 0.00 | 0.545 | 1.21 | 1.53 | 1.75 | 2.33 | 1.93 | 1.71 | 1.61 | 1.48 | 1.37 | 1.34 | 1.32 | 1.31 | 1.06 | 0.813 | 0.672 | 0.496 | 0.365 | 0.242 | 0.209 | 0.143 |
| K017 | TR | 2 | TR | R | | 0.00 | 0.415 | 1.11 | 1.58 | 2.43 | 3.04 | 3.51 | 3.28 | 3.02 | 3.10 | 3.07 | 3.21 | 2.99 | 2.75 | 2.29 | 1.90 | 1.32 | 0.742 | 0.402 | 0.219 | 0.127 | 0.0827 |
| K018 | RT | 1 | RT | R | | 0.00 | 0.839 | 1.87 | 2.11 | 1.95 | 2.37 | 2.46 | 2.64 | 2.46 | 2.30 | 2.11 | 2.21 | 1.97 | 1.97 | 1.73 | 1.44 | 1.18 | 0.855 | 0.619 | 0.463 | 0.308 | 0.254 |
| K019 | TR | 2 | TR | R | | 0.00 | 0.147 | 0.618 | 0.607 | 1.70 | 3.77 | 3.11 | 3.21 | 2.81 | 2.74 | 2.39 | 2.30 | 2.30 | 2.06 | 1.69 | 1.57 | 1.22 | 0.865 | 0.693 | 0.505 | 0.381 | 0.307 |
| K020 | RT | 1 | RT | R | | 0.00 | 0.593 | 1.17 | 1.41 | 1.48 | 2.14 | 2.62 | 2.35 | 2.34 | 2.14 | 2.24 | 2.45 | 2.32 | 1.97 | 1.86 | 1.72 | 1.39 | 0.988 | 0.665 | 0.428 | 0.305 | 0.265 |
| K021 | TR | 2 | TR | R | | 0.00 | 0.251 | 1.12 | 1.47 | 1.32 | 2.32 | 2.63 | 2.31 | 1.82 | 1.94 | 1.76 | 1.65 | 1.95 | 1.86 | 1.69 | 1.48 | 1.25 | 0.830 | 0.737 | 0.419 | 0.294 | 0.239 |
| K022 | TR | 2 | TR | R | | 0.00 | 0.307 | 0.497 | 1.06 | 1.38 | 2.10 | 2.23 | 2.24 | 2.29 | 2.25 | 2.14 | 2.55 | 2.37 | 2.20 | 1.89 | 1.62 | 1.31 | 0.903 | 0.841 | 0.558 | 0.452 | 0.298 |
| K023 | RT | 1 | RT | R | | 0.00 | 0.561 | 1.02 | 1.59 | 1.34 | 2.33 | 2.02 | 1.93 | 1.96 | 2.08 | 1.79 | 2.04 | 2.01 | 1.82 | 1.45 | 1.20 | 0.971 | 0.695 | 0.469 | 0.331 | 0.195 | 0.136 |
| K024 | RT | 1 | RT | R | | 0.00 | 0.593 | 1.52 | 2.25 | 2.31 | 2.63 | 2.54 | 2.23 | 2.48 | 2.58 | 2.36 | 2.45 | 2.47 | 2.26 | 1.96 | 1.69 | 1.22 | 0.899 | 0.656 | 0.466 | 0.361 | 0.261 |
| Number of valid data | | | | | 24 | 24 | 24 | 24 | 24 | 24 | 24 | 24 | 24 | 24 | 24 | 24 | 24 | 24 | 24 | 24 | 24 | 24 | 24 | 24 | 24 | 24 | 24 |
| Mean concentration | | | | | 0.238 | / | 0.648 | 1.30 | 1.59 | 1.82 | 2.56 | 2.64 | 2.56 | 2.39 | 2.34 | 2.17 | 2.22 | 2.14 | 2.08 | 1.77 | 1.52 | 1.22 | 0.841 | 0.634 | 0.428 | 0.316 | 0.238 |
| SD | | | | | 0.0978 | / | 0.397 | 0.501 | 0.532 | 0.538 | 0.502 | 0.511 | 0.545 | 0.477 | 0.484 | 0.482 | 0.463 | 0.450 | 0.435 | 0.391 | 0.374 | 0.277 | 0.217 | 0.186 | 0.147 | 0.122 | 0.0978 |
| CV% | | | | | / | | / | 38.5 | 33.5 | 29.6 | 19.6 | 19.3 | 21.3 | 20.0 | 20.7 | 22.3 | 20.9 | 21.1 | 21.0 | 22.1 | 24.6 | 22.7 | 25.8 | 29.3 | 34.4 | 38.7 | 41.0 |
| Maximum | | | | | / | | / | 2.24 | 2.74 | 3.22 | 3.77 | 3.73 | 3.50 | 3.21 | 3.25 | 3.07 | 3.21 | 2.99 | 3.20 | 2.52 | 2.48 | 1.86 | 1.40 | 0.983 | 0.754 | 0.594 | 0.454 |
| Minimum | | | | | / | | / | 0.497 | 0.607 | 1.11 | 1.70 | 1.78 | 1.71 | 1.61 | 1.48 | 1.37 | 1.34 | 1.32 | 1.31 | 1.06 | 0.813 | 0.672 | 0.469 | 0.330 | 0.177 | 0.127 | 0.0827 |

The concentration lower than the lower limit of quantification before T_max_ was expressed as 0. The concentration lower than the lower limit of quantification after T_max_ was expressed as deletion (ND) in PK analysis and 0 in descriptive statistics.

**The High Fat Meal Group**

**Table 3.** Individual plasma concentration-time data of levamlodipine test formulation in the high fat meal group (ng/ml). (Supplementary file for Fig. 3)

| SUB | SEQ | PER | GRP | TRT | C1 | C2 | C3 | C4 | C5 | C6 | C7 | C8 | C9 | C10 | C11 | C12 | C13 | C14 | C15 | C16 | C17 | C18 | C19 | C20 | C21 | C22 |
| --- | --- | --- | --- | --- | --- | --- | --- | --- | --- | --- | --- | --- | --- | --- | --- | --- | --- | --- | --- | --- | --- | --- | --- | --- | --- | --- |
| F001 | RT | 2 | RT | T | 0.00 | 0.568 | 1.25 | 1.53 | 2.53 | 3.37 | 3.74 | 3.49 | 3.35 | 3.38 | 3.36 | 3.16 | 2.80 | 3.01 | 2.76 | 2.51 | 1.91 | 1.43 | 1.00 | 0.728 | 0.571 | 0.416 |
| F002 | TR | 1 | TR | T | 0.00 | 0.275 | 0.979 | 1.31 | 1.61 | 2.65 | 2.81 | 3.09 | 2.66 | 3.21 | 2.48 | 2.82 | 2.46 | 2.11 | 1.95 | 1.68 | 1.38 | 0.843 | 0.506 | 0.401 | 0.292 | 0.201 |
| F003 | RT | 2 | RT | T | 0.00 | 0.274 | 0.644 | 1.02 | 1.18 | 1.39 | 1.41 | 1.44 | 1.69 | 1.89 | 2.06 | 2.29 | 2.14 | 2.51 | 1.82 | 1.64 | 1.12 | 0.846 | 0.601 | 0.450 | 0.309 | 0.241 |
| F004 | TR | 1 | TR | T | 0.00 | 0.672 | 1.10 | 1.90 | 2.09 | 2.93 | 2.89 | 3.27 | 3.09 | 3.57 | 3.19 | 3.57 | 3.74 | 3.04 | 3.02 | 2.83 | 2.48 | 2.05 | 1.80 | 1.54 | 1.27 | 1.05 |
| F005 | RT | 2 | RT | T | 0.00 | 0.483 | 0.994 | 1.14 | 1.19 | 1.56 | 1.88 | 1.82 | 1.78 | 1.86 | 1.80 | 1.67 | 1.59 | 1.64 | 1.42 | 1.32 | 1.07 | 0.860 | 0.669 | 0.432 | 0.408 | 0.310 |
| F006 | TR | 1 | TR | T | 0.00 | 0.169 | 0.680 | 1.14 | 1.16 | 2.09 | 2.47 | 2.40 | 2.73 | 2.56 | 2.38 | 2.59 | 2.36 | 2.23 | 1.89 | 1.56 | 1.38 | 0.804 | 0.613 | 0.399 | 0.299 | 0.198 |
| F007 | RT | 2 | RT | T | 0.00 | 0.149 | 0.543 | 1.24 | **1.81^** | 2.63 | 2.81 | 3.17 | 2.87 | 2.77 | 2.91 | 3.12 | 2.84 | 3.16 | 2.23 | 2.33 | 1.56 | 1.13 | 0.758 | 0.527 | 0.399 | 0.239 |
| F008 | TR | 1 | TR | T | 0.00 | 0.799 | 2.12 | 2.21 | 1.91 | 3.01 | 2.59 | 2.68 | 2.34 | 2.60 | 2.58 | 2.39 | 2.38 | 2.11 | 1.98 | 1.73 | 1.42 | 1.01 | 0.708 | 0.550 | 0.389 | 0.287 |
| F009 | RT | 2 | RT | T | 0.00 | 1.75 | 3.36 | 3.10 | 2.64 | 3.73 | 3.81 | 3.71 | 3.37 | 3.44 | 3.17 | 3.41 | 3.24 | 3.72 | 2.78 | 2.45 | 1.95 | 1.42 | 1.02 | 0.746 | 0.466 | 0.321 |
| F010 | RT | 2 | RT | T | 0.00 | 0.793 | 1.95 | 2.23 | 1.26 | 1.77 | 2.26 | 2.24 | 2.11 | 2.01 | 1.76 | 1.94 | 1.77 | 2.16 | 1.61 | 1.31 | 1.09 | 0.792 | 0.434 | 0.303 | 0.209 | 0.135 |
| F011 | TR | 1 | TR | T | 0.00 | 0.194 | 1.07 | 1.40 | 1.49 | 2.53 | 2.39 | 2.49 | 2.25 | 2.12 | 1.87 | 1.95 | 1.57 | 1.66 | 1.61 | 1.34 | 1.14 | 0.754 | 0.596 | 0.416 | 0.322 | 0.244 |
| F012 | TR | 1 | TR | T | 0.00 | 0.570 | 1.58 | 1.64 | 1.75 | 2.59 | 2.18 | 2.27 | 2.13 | 2.03 | 1.68 | 1.70 | 1.71 | 1.45 | 1.28 | 0.999 | 0.840 | 0.627 | 0.534 | 0.380 | 0.297 | 0.211 |
| F013 | RT | 2 | RT | T | 0.00 | 0.0678 | 0.0878 | 0.145 | 0.222 | 0.604 | 1.18 | 1.53 | 2.24 | 2.16 | 2.13 | 2.54 | 2.24 | 2.74 | 2.02 | 1.61 | 1.22 | 0.892 | 0.675 | 0.451 | 0.359 | 0.242 |
| F014 | TR | 1 | TR | T | 0.00 | 0.0553 | 0.390 | 1.13 | 1.35 | 2.71 | 3.16 | 3.22 | 2.96 | 3.01 | 2.34 | 2.25 | 2.46 | 2.22 | 1.88 | 1.49 | 1.17 | 0.822 | 0.517 | 0.342 | 0.228 | 0.168 |
| F015 | TR | 1 | TR | T | 0.00 | 0.00 | 0.0758 | 0.137 | 0.210 | 0.745 | 1.40 | 1.68 | 2.10 | 2.21 | 1.94 | 2.11 | 2.08 | 2.01 | 1.80 | 1.66 | 1.46 | 0.980 | 0.746 | 0.480 | 0.379 | 0.294 |
| F016 | RT | 2 | RT | T | 0.00 | 0.00 | 0.0561 | 0.106 | 0.186 | 0.620 | 1.30 | 2.45 | 2.67 | 2.85 | 2.87 | 3.06 | 2.91 | 2.73 | 2.48 | 2.16 | 1.64 | 1.29 | 0.936 | 0.661 | 0.483 | 0.379 |
| F017 | RT | 2 | RT | T | 0.00 | 0.531 | 1.16 | 1.10 | 1.21 | 1.64 | 1.78 | 2.20 | 2.19 | 2.30 | 2.31 | 2.34 | 2.32 | 2.07 | 1.94 | 1.45 | 1.14 | 0.720 | 0.430 | 0.283 | 0.191 | 0.125 |
| F018 | TR | 1 | TR | T | 0.00 | 0.00 | 0.310 | 0.982 | 1.01 | 1.67 | **1.91^** | 1.89 | 1.69 | 1.86 | 1.92 | 1.93 | 2.00 | 1.78 | 1.92 | 1.48 | 1.22 | 0.776 | 0.570 | 0.389 | 0.309 | 0.235 |
| F019 | TR | 1 | TR | T | 0.00 | 0.00 | 0.139 | 0.277 | 0.473 | 1.06 | 1.56 | 2.22 | 2.19 | 2.05 | 2.16 | 2.34 | 2.26 | 1.85 | 1.77 | 1.55 | 1.35 | 1.02 | 0.752 | 0.549 | 0.421 | 0.353 |
| F020 | RT | 2 | RT | T | 0.00 | 0.00 | 0.133 | 0.285 | 0.557 | 1.10 | 1.72 | 2.41 | 2.41 | 2.42 | 2.36 | 2.59 | 2.50 | 2.10 | 1.60 | 1.52 | 1.06 | 0.753 | 0.624 | 0.470 | 0.363 | 0.261 |
| F021 | RT | 2 | RT | T | 0.00 | 0.236 | 0.725 | 1.48 | 1.33 | 2.25 | 2.64 | 2.07 | 2.05 | 2.11 | 2.01 | 2.13 | 2.12 | 2.15 | 1.61 | 1.45 | 1.21 | 0.923 | 0.819 | 0.614 | 0.465 | 0.358 |
| F022 | TR | 1 | TR | T | 0.00 | 0.413 | 1.30 | 1.65 | 1.58 | 2.20 | 2.19 | 2.20 | 2.15 | 1.94 | 1.81 | 1.97 | 1.83 | 1.65 | 1.58 | 1.38 | 1.21 | 0.819 | 0.581 | 0.413 | 0.294 | 0.180 |
| F023 | RT | 2 | RT | T | 0.00 | 0.00 | 0.253 | 0.522 | 1.72 | 1.95 | 2.52 | 2.79 | 2.46 | 2.15 | 2.01 | 1.75 | 1.91 | 1.90 | 1.77 | 1.49 | 1.14 | 1.11 | 0.725 | 0.571 | 0.389 | 0.287 |
| F024 | TR | 1 | TR | T | 0.00 | 0.157 | 0.411 | 0.638 | 0.903 | 1.68 | 2.14 | 1.89 | 1.80 | 2.04 | 1.63 | 1.66 | 1.53 | 1.58 | 1.24 | 0.993 | 0.862 | 0.524 | 0.354 | 0.198 | 0.142 | 0.0791 |
| Mean concentration | | | | | 24 | 24 | 24 | 24 | 23 | 24 | 23 | 24 | 24 | 24 | 24 | 24 | 24 | 24 | 24 | 24 | 24 | 24 | 24 | 24 | 24 | 24 |
| SD | | | | | / | 0.340 | 0.888 | 1.18 | 1.29 | 2.02 | 2.30 | 2.44 | 2.39 | 2.44 | 2.28 | 2.39 | 2.28 | 2.23 | 1.92 | 1.66 | 1.33 | 0.966 | 0.707 | 0.512 | 0.386 | 0.284 |
| CV% | | | | | / | 0.399 | 0.788 | 0.746 | 0.675 | 0.857 | 0.723 | 0.623 | 0.486 | 0.541 | 0.504 | 0.559 | 0.544 | 0.574 | 0.456 | 0.464 | 0.368 | 0.323 | 0.288 | 0.257 | 0.213 | 0.183 |
| Maximum | | | | | / | 118 | 88.8 | 63.3 | 52.5 | 42.4 | 31.5 | 25.5 | 20.4 | 22.2 | 22.1 | 23.4 | 23.8 | 25.7 | 23.8 | 27.9 | 27.6 | 33.4 | 40.7 | 50.1 | 55.2 | 64.4 |
| Minimum | | | | | / | 1.75 | 3.36 | 3.10 | 2.64 | 3.73 | 3.81 | 3.71 | 3.37 | 3.57 | 3.36 | 3.57 | 3.74 | 3.72 | 3.02 | 2.83 | 2.48 | 2.05 | 1.80 | 1.54 | 1.27 | 1.05 |
| Mean concentration | | | | | / | 0.00 | 0.0561 | 0.106 | 0.186 | 0.604 | 1.18 | 1.44 | 1.69 | 1.86 | 1.63 | 1.66 | 1.53 | 1.45 | 1.24 | 0.993 | 0.840 | 0.524 | 0.354 | 0.198 | 0.142 | 0.0791 |

**Table 4.** Individual plasma concentration-time data of levamlodipine reference formulation in the high fat meal group (ng/ml). (Supplementary file for Fig. 3)

| SUB | SEQ | PER | GRP | TRT | C1 | C2 | C3 | C4 | C5 | C6 | C7 | C8 | C9 | C10 | C11 | C12 | C13 | C14 | C15 | C16 | C17 | C18 | C19 | C20 | C21 | C22 |
| --- | --- | --- | --- | --- | --- | --- | --- | --- | --- | --- | --- | --- | --- | --- | --- | --- | --- | --- | --- | --- | --- | --- | --- | --- | --- | --- |
| F001 | RT | 1 | RT | R | 0.00 | 3.55 | 5.47 | 5.08 | 3.86 | 4.13 | 4.03 | 3.58 | 3.42 | 3.46 | 3.18 | 3.11 | 2.83 | 2.80 | 2.83 | 2.24 | 2.03 | 1.28 | 0.944 | 0.648 | 0.488 | 0.375 |
| F002 | TR | 2 | TR | R | 0.00 | 0.606 | 2.18 | 1.21 | 1.79 | 3.35 | 3.04 | 2.97 | 2.77 | 2.63 | 2.69 | 2.72 | 2.29 | 2.46 | 2.01 | 1.51 | 1.29 | 0.863 | 0.624 | 0.420 | 0.291 | 0.216 |
| F003 | RT | 1 | RT | R | 0.00 | 0.616 | 1.80 | 1.70 | 1.34 | 2.30 | 2.01 | 2.10 | 1.93 | 2.00 | 1.73 | 1.84 | 1.82 | 1.68 | 1.74 | 1.36 | 1.20 | 0.751 | 0.542 | 0.365 | 0.292 | 0.206 |
| F004 | TR | 2 | TR | R | 0.105 | 0.132 | 0.183 | 0.454 | 0.506 | 1.50 | 1.92 | 2.31 | 2.64 | 2.59 | 2.78 | 3.12 | 2.75 | 3.02 | 2.56 | 2.62 | 2.18 | 1.75 | 1.58 | 1.37 | 1.06 | 0.979 |
| F005 | RT | 1 | RT | R | 0.00 | 0.630 | 1.10 | 2.06 | 1.68 | 2.28 | 1.98 | 2.03 | 1.96 | 1.78 | 1.64 | 1.68 | 1.74 | 1.58 | 1.26 | 1.13 | 0.980 | 0.693 | 0.630 | 0.445 | 0.340 | 0.250 |
| F006 | TR | 2 | TR | R | 0.00 | 0.0617 | 0.0806 | 0.451 | 0.721 | 1.80 | 1.99 | 2.34 | 2.23 | 2.75 | 2.86 | 2.55 | 2.46 | 2.44 | 1.90 | 1.54 | 1.15 | 0.844 | 0.580 | 0.380 | 0.293 | 0.178 |
| F007 | RT | 1 | RT | R | 0.00 | 0.0698 | 0.607 | 0.783 | 1.14 | 2.42 | 2.42 | 2.65 | 2.48 | 3.31 | 2.90 | 3.38 | 3.03 | 2.81 | 2.10 | 1.99 | 1.54 | 0.930 | 0.724 | 0.520 | 0.388 | 0.267 |
| F008 | TR | 2 | TR | R | 0.00 | 1.85 | 4.11 | 2.88 | 2.46 | 3.16 | 2.90 | 2.86 | 2.60 | 2.42 | 2.34 | 2.37 | 2.29 | 2.46 | 1.98 | 1.81 | 1.49 | 1.18 | 0.856 | 0.553 | 0.395 | 0.341 |
| F009 | RT | 1 | RT | R | 0.00 | 0.754 | 2.26 | 2.96 | 2.86 | 3.73 | 3.91 | 3.86 | 3.88 | 4.08 | 3.95 | 3.83 | 3.64 | 3.55 | 2.74 | 2.63 | 2.11 | 1.41 | 0.965 | 0.697 | 0.500 | 0.418 |
| F010 | RT | 1 | RT | R | 0.00 | 0.483 | 1.01 | 1.42 | 1.46 | 1.65 | 2.18 | 1.80 | 2.07 | 2.18 | 1.90 | 1.72 | 1.99 | 1.68 | 1.56 | 1.26 | 1.06 | 0.689 | 0.490 | 0.295 | 0.157 | 0.139 |
| F011 | TR | 2 | TR | R | 0.00 | 0.0770 | 0.181 | 0.281 | 0.459 | 1.99 | 2.38 | 2.50 | 2.13 | 2.05 | 1.87 | 1.73 | 1.86 | 1.78 | 1.47 | 1.18 | 0.882 | 0.657 | 0.478 | 0.320 | 0.229 | 0.153 |
| F012 | TR | 2 | TR | R | 0.00 | 0.0788 | 0.538 | 1.90 | 2.12 | 2.61 | 2.93 | 3.01 | 2.55 | 2.52 | 2.33 | 2.05 | 1.96 | 2.24 | 1.49 | 1.30 | 1.11 | 0.831 | 0.687 | 0.425 | 0.362 | 0.241 |
| F013 | RT | 1 | RT | R | 0.00 | 0.385 | 0.648 | 1.32 | 1.20 | 2.05 | 2.47 | 2.30 | 2.06 | 1.73 | 2.14 | 2.14 | 2.37 | 2.06 | 1.82 | 1.43 | 1.18 | 0.720 | 0.556 | 0.380 | 0.376 | 0.260 |
| F014 | TR | 2 | TR | R | 0.00 | 0.0900 | 0.682 | 1.44 | 1.71 | 2.69 | 3.24 | 3.00 | 2.49 | 2.76 | 2.65 | 2.55 | 2.64 | 2.38 | 1.95 | 1.67 | 1.14 | 0.723 | 0.506 | 0.319 | 0.242 | 0.141 |
| F015 | TR | 2 | TR | R | 0.00 | 0.347 | 1.47 | 1.68 | 1.74 | 1.88 | 2.48 | 2.59 | 2.38 | 2.53 | 2.63 | 2.77 | 2.85 | 2.33 | 2.32 | 2.12 | 1.53 | 1.23 | 1.02 | 0.631 | 0.495 | 0.368 |
| F016 | RT | 1 | RT | R | 0.00 | 1.00 | 2.04 | 2.40 | 2.46 | 2.06 | 2.54 | 2.56 | 2.44 | 2.48 | 2.08 | 2.20 | 2.30 | 2.29 | 2.21 | 1.76 | 1.50 | 0.928 | 0.719 | 0.481 | 0.375 | 0.286 |
| F017 | RT | 1 | RT | R | 0.00 | 0.120 | 0.545 | 0.902 | 1.23 | 2.19 | 2.35 | 2.27 | 2.32 | 2.39 | 2.08 | 2.21 | 2.27 | 2.01 | 2.04 | 1.61 | 1.32 | 0.789 | 0.472 | 0.311 | 0.204 | 0.146 |
| F018 | TR | 2 | TR | R | 0.00 | 0.00 | 0.00 | 0.0839 | 0.221 | 0.495 | 0.740 | 1.05 | 1.02 | 1.19 | 1.44 | 1.30 | 1.61 | 1.61 | 1.74 | 1.51 | 1.17 | 0.798 | 0.534 | 0.362 | 0.252 | 0.197 |
| F019 | TR | 2 | TR | R | 0.00 | 0.309 | 1.22 | 1.40 | 2.36 | 2.17 | 1.82 | 2.12 | 1.90 | 2.07 | 1.78 | 1.87 | 1.98 | 2.06 | 1.91 | 1.45 | 1.27 | 0.955 | 0.773 | 0.507 | 0.397 | 0.328 |
| F020 | RT | 1 | RT | R | 0.00 | 0.123 | 0.486 | 0.621 | 0.933 | 1.94 | 1.98 | 2.20 | 2.41 | 2.64 | 2.49 | 2.44 | 2.43 | 2.05 | 1.64 | 1.50 | 1.24 | 0.732 | 0.536 | 0.382 | 0.309 | 0.251 |
| F021 | RT | 1 | RT | R | 0.00 | 0.897 | 1.81 | 1.88 | 1.63 | 1.80 | 1.90 | 2.10 | 2.05 | 2.07 | 2.00 | 1.77 | 1.83 | 1.63 | 1.57 | 1.25 | 1.13 | 0.815 | 0.595 | 0.524 | 0.401 | 0.314 |
| F022 | TR | 2 | TR | R | 0.00 | 2.77 | 2.98 | 3.19 | 2.87 | 2.51 | 2.88 | 2.32 | 2.22 | 2.42 | 2.11 | 2.12 | 2.03 | 1.92 | 1.72 | 1.61 | 1.25 | 0.882 | 0.691 | 0.496 | 0.318 | 0.250 |
| F023 | RT | 1 | RT | R | 0.00 | 0.286 | 1.14 | 2.17 | 1.29 | 2.43 | 2.16 | 1.84 | 1.70 | 1.86 | 1.64 | 1.65 | 1.64 | 1.60 | 1.39 | 1.26 | 1.19 | 0.752 | 0.593 | 0.440 | 0.295 | 0.218 |
| F024 | TR | 2 | TR | R | 0.00 | 0.00 | 0.00 | 0.134 | 0.690 | 1.48 | 2.03 | 2.06 | 2.20 | 2.10 | 1.98 | 2.03 | 2.06 | 2.15 | 1.77 | 1.49 | 1.13 | 0.937 | **0.623^** | 0.357 | 0.279 | 0.169 |
| Mean concentration | | | | | 24 | 24 | 24 | 24 | 24 | 24 | 24 | 24 | 24 | 24 | 24 | 24 | 24 | 24 | 24 | 24 | 24 | 24 | 23 | 24 | 24 | 24 |
| SD | | | | | / | 0.635 | 1.36 | 1.60 | 1.61 | 2.28 | 2.43 | 2.43 | 2.33 | 2.42 | 2.30 | 2.30 | 2.28 | 2.19 | 1.91 | 1.63 | 1.34 | 0.922 | 0.700 | 0.485 | 0.364 | 0.279 |
| CV% | | | | | / | 0.892 | 1.34 | 1.16 | 0.881 | 0.769 | 0.701 | 0.589 | 0.550 | 0.605 | 0.580 | 0.616 | 0.493 | 0.502 | 0.405 | 0.416 | 0.340 | 0.266 | 0.249 | 0.218 | 0.173 | 0.168 |
| Maximum | | | | | / | 141 | 99.0 | 72.3 | 54.6 | 33.8 | 28.9 | 24.2 | 23.6 | 25.1 | 25.2 | 26.8 | 21.6 | 22.9 | 21.3 | 25.4 | 25.4 | 28.9 | 35.6 | 45.0 | 47.5 | 60.4 |
| Minimum | | | | | / | 0.105 | 3.55 | 5.47 | 5.08 | 3.86 | 4.13 | 4.03 | 3.86 | 3.88 | 4.08 | 3.95 | 3.83 | 3.64 | 3.55 | 2.83 | 2.63 | 2.18 | 1.75 | 1.58 | 1.37 | 1.06 |
| Mean concentration | | | | | / | 0.00 | 0.00 | 0.00 | 0.0839 | 0.221 | 0.495 | 0.740 | 1.05 | 1.02 | 1.19 | 1.44 | 1.30 | 1.61 | 1.58 | 1.26 | 1.13 | 0.882 | 0.657 | 0.472 | 0.295 | 0.157 |
